# Comprehensive Deciphering the Complexity of the Deep Bite: Insight from Animal Model to Human Subjects

**DOI:** 10.3390/jpm13101472

**Published:** 2023-10-08

**Authors:** Nezar Watted, Iqbal M. Lone, Osayd Zohud, Kareem Midlej, Peter Proff, Fuad A. Iraqi

**Affiliations:** 1 Center for Dentistry Research and Aesthetics, Jatt 45911, Israel; nezar.watted@gmx.net; 2 Department of Orthodontics, Faculty of Dentistry, Arab America University, Jenin 919000, Palestine; 3 Gathering for Prosperity Initiative, Jatt 45911, Israel; 4 Department of Clinical Microbiology and Immunology, Sackler Faculty of Medicine, Tel-Aviv University, Tel Aviv 69978, Israel; iqbalzoo84@gmail.com (I.M.L.); osaydzohud@mail.tau.ac.il (O.Z.);; 5 University Hospital of Regensburg, Department of Orthodontics, University of Regensburg, 93053 Regensburg, Germany

**Keywords:** deep bite, clinical treatment strategies, genetics of deep bite, animal model, genomics approaches

## Abstract

Deep bite is a malocclusion phenotype, defined as the misalignment in the vertical dimension of teeth and jaws and characterized by excessive overlap of the upper front teeth over the lower front teeth. Numerous factors, including genetics, environmental factors, and behavioral ones, might contribute to deep bite. In this study, we discuss the current clinical treatment strategies for deep bite, summarize the already published findings of genetic analysis associated with this complex phenotype, and their constraints. Finally, we propose a comprehensive roadmap to facilitate investigations for determining the genetic bases of this complex phenotype development. Initially, human deep bite phenotype, genetics of human deep bite, the prevalence of human deep bite, diagnosis, and treatment of human deep bite were the search terms for published publications. Here, we discuss these findings and their limitations and our view on future strategies for studying the genetic bases of this complex phenotype. New preventative and treatment methods for this widespread dental issue can be developed with the help of an understanding of the genetic and epigenetic variables that influence malocclusion. Additionally, malocclusion treatment may benefit from technological developments like 3D printing and computer-aided design and manufacture (CAD/CAM). These technologies enable the development of personalized surgical and orthodontic guidelines, enhancing the accuracy and effectiveness of treatment. Overall, the most significant results for the patient can only be achieved with a customized treatment plan created by an experienced orthodontic professional. To design a plan that meets the patient’s specific requirements and expectations, open communication between the patient and the orthodontist is essential. Here, we propose to conduct a genome-wide association study (GWAS), RNAseq analysis, integrating GWAS and expression quantitative trait loci (eQTL), micro and small RNA, and long noncoding RNA analysis in tissues associated with deep bite malocclusion in human, and complement it by the same approaches in the collaborative cross (CC) mouse model which offer a novel platform for identifying genetic factors as a cause of deep bite in mice, and subsequently can then be translated to humans. An additional direct outcome of this study is discovering novel genetic elements to advance our knowledge of how this malocclusion phenotype develops and open the venue for early identification of patients carrying the susceptible genetic factors so that we can offer early prevention and treatment strategies, a step towards applying a personalized medicine approach.

## 1. Introduction

The term “malocclusion”, which refers to the misalignment of upper and lower teeth and jaws and is a widespread dental condition affecting millions of people worldwide, can negatively affect oral health, including limiting function such as the ability to eat, speak, and practice good oral hygiene [1]. Malocclusion can also affect how one looks and lead to a loss of confidence and self-esteem [1]. Genetic, environmental, and developmental factors are among malocclusion’s multifactorial and complex causes [2]. Dental research has made significant strides toward understanding the molecular causes of malocclusion, with genetics and genomics shedding light on particular genes and signaling pathways that affect jaw development, tooth eruption, and completion of tooth root development. It is still difficult to fully comprehend the complex interactions between genetic and environmental factors that lead to malocclusion [3].

**Figure 1:**
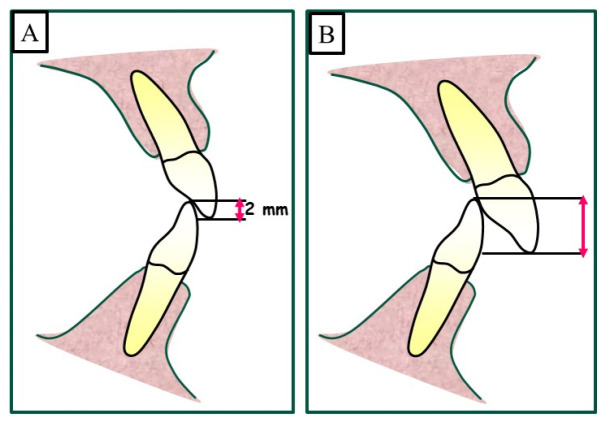
Definition of an overbite as a vertical relationship or the distance between the maxillary central incisor and the opposing mandibular central incisor. (**A**) shows a physiological overbite of 2–3 mm, and a deep bite caused when overbite increased by more than 3 mm is shown in (**B**).

A deep bite is a common orthodontic problem when the upper front teeth overlap the lower front teeth excessively (Figure 1). This condition can cause various issues, including difficulty in the function of biting and chewing, speech problems, and even jaw pain.

**Figure 2:**
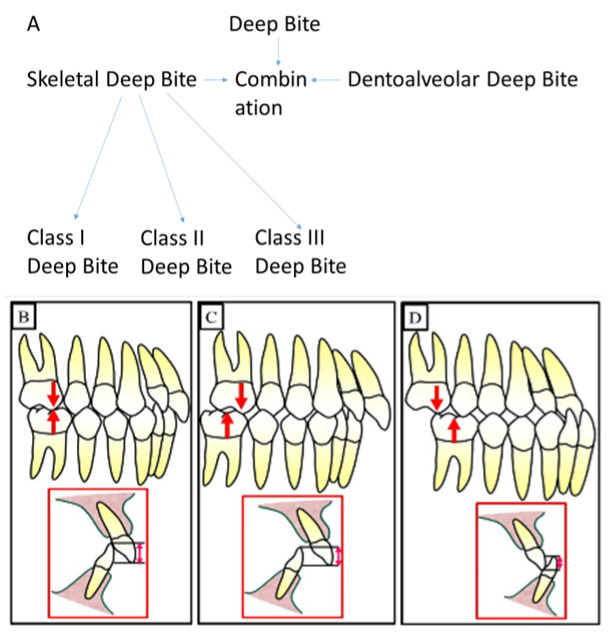
The different types of deep bite. (**A**) shows a diagram to illustrate the different types of deep bites. (**B**) shows a deep bite with Class I. (**C**) shows deep bite with Class II, and (**D**) shows a deep bite with Class III.

The deep bite can be underlined and defined by different types including skeletal deep bite, dentoaleolar deep bite, and a combination of skeletal and dentoalveolar deep bite (Figure 2).

**Figure 3:**
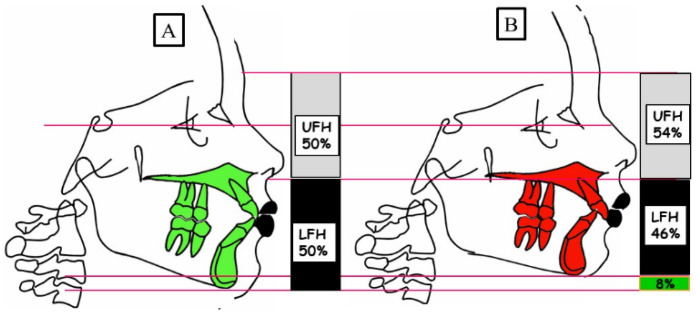
Schematic representation of the vertical dimension for a physiological overbite (**A**) and skeletal deep bite (**B**). (**A**) Physiological vertical dimension; harmonious relation between the upper facial height (UFH 50%) and lower facial height (LFH 50%). (**B**) A skeletal deep bite because of the anterior rotation of the mandible. There is a decreased lower facial height (LFH 46%) compared to the upper face height (UFH 54%).

It is known that the type of skeletal deep bite is caused by growth disturbance in the vertical dimension and is called anterior growth. The interbasal angle between the base of the maxilla and the base of the mandible decreases due to the anterior rotation of the mandible during growth. Accordingly, the soft tissue structures develop and adapt to “soft tissue go with the bone”. The result as a phenotype is “***short face syndrome****”*. The skeletal deep bite can occur in all angle classes, as shown in Figure 3.

**Figure 4:**
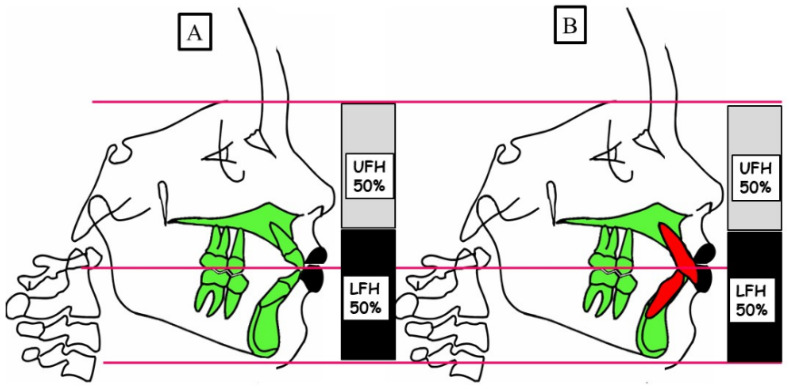
Schematic representation of the vertical dimension for a physiological overbite (**A**) and dentoalveolar deep bite (**B**). (**A**) Physiological vertical dimension; harmonious relation between the upper facial height (UFH 50%) and lower facial height (LFH 50%). (**B**) A dentoalveolar deep bite because of the elongation of the front teeth; harmonious relation between the upper facial height (UFH 50%) and lower facial height (LFH 50%).

The deep bite defined by dental malformation is caused in the vertical dimension. Elongation, teeth that usually erupted too far, or tooth migration result in a significant vertical overlap of the front teeth. This is usually not associated with profile changes in the vertical dimension, as shown in Figure 4.

**Figure 5:**
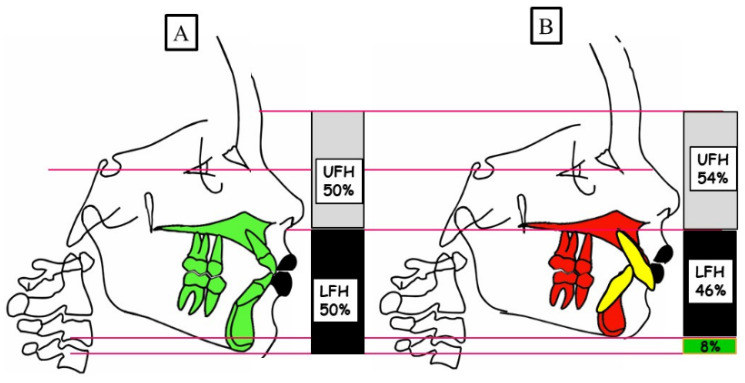
Schematic representation of the vertical dimension for a physiological overbite (**A**) and the combination of skeletal and dentoalveolar deep bite (**B**). (**A**) Physiological vertical dimension; harmonious relation between the upper facial height (UFH 50%) and lower facial height (LFH 50%). (**B**) A skeletal and dentoalveolar deep bite because of the anterior rotation of the mandible and elongation of the front teeth. There is a decreased lower facial height (LFH 46%) compared to the upper face height (UFH 54%).

Finally, the deep bite may be caused by a combination of skeletal and dentoalveolar dysgnathy. In this case, the patient’s phenotype and profile exhibits a disharmony in vertical dimension “*short face syndrome”*, as shown in Figure 5.

A deep bite can be caused by various factors, including genetics, environmental and behavioral factors [4]. While environmental factors such as thumb sucking, tongue-thrusting, and prolonged pacifier use can contribute to the development of deep bites, there is also evidence to suggest that genetics may play a role [4]. Several studies have identified specific genes that may be associated with the development of deep bite. Many researchers have identified specific genes that may be associated with the development of deep bite, such as the IRF6 and BMP4 genes [2,5]. Other studies have suggested that variations in genes related to bone growth and development, such as the RUNX2 and COL1A1 genes, may also play a role in the development of deep bite [6]. Overall, while environmental factors can contribute to the development of deep bite, there is evidence to suggest that genetics may also play a vital role. Further research is needed to identify specific genes and genetic pathways that are involved in the development of this malocclusion.

With the aid of animal models, the molecular analysis of malocclusion has been dramatically enhanced. With the help of these models, researchers can examine how environmental and genetic factors affect the development of misaligned teeth and test various preventative and therapeutic strategies. Because of their small size, simplicity in breeding, and genetic resemblance to humans, mice are one of the most commonly used animal models [7]. The collaborative cross (CC) mouse is an effective tool for researching complex genetic traits like malocclusion. CC mice are bred to produce a genetically diverse population to increase their genetic variation. This mouse population is a great model for research on the genetic causes of malocclusion because it has a distinctive and varied array of genetic variants [7]. The CC mouse population has previously been used to study complex genetic traits like body weight, and metabolic disorders [8]. The genetic diversity of CC mice offers a powerful tool to comprehend the genetic components of malocclusion and enables researchers to pinpoint genetic variants that contribute to complex traits [9,10].

### 1.1. Etiology

Epigenetic factors, such as DNA methylation, histone modification, and microRNA regulation, can also play a role in malocclusion development. Epigenetic changes can alter gene expression patterns and influence the development of the teeth and jaws. For example, studies have shown that DNA methylation patterns are altered in patients with malocclusion, and these changes can affect gene expression patterns involved in jaw growth and tooth eruption [11]. Additionally, epigenetic changes can be influenced by environmental factors such as diet, stress, and exposure to toxins. For example, studies have shown that maternal stress during pregnancy can alter DNA methylation patterns in the offspring, potentially leading to craniofacial abnormalities and malocclusion [12].

In summary, the genetic causes of malocclusion involve variations in genes and signaling pathways that are involved in jaw growth, tooth eruption, and dental occlusion. Epigenetic factors, such as DNA methylation and microRNA regulation, can also play a role in malocclusion development. Understanding the genetic and epigenetic factors that contribute to malocclusion can inform the development of new prevention and treatment strategies for this common dental problem.

There are numerous unnoticed skeletal or dental irregularities under a deep bite malocclusion. Therefore, it is essential to understand that a deep bite is not a disease, but rather a clinical expression of an underlying skeletal or dental irregularity. Skeletal or dental overbites are influenced by environmental, genetic, or a mix of environmental and genetic variables during development. Skeletal deep bites are typically characterized by (1) a growing mismatch between the mandibular and maxillary jawbones, (2) convergent rotation of the jaw bases, and/or (3) a deficient mandibular ramus height. Particularly in the lower portion of the face, the anterior facial height is frequently tiny in such circumstances. The incisors’ supraocclusion (overeruption), on the other hand, is indicated by dental deep bites [13] or infraocclusion (undereruption) of the molars or a combination of the two [14]. Alterations to the tooth morphologies, early loss of permanent teeth leading to a lingual collapse of the maxillary or mandibular anterior teeth, mesiodistal breadth of anterior teeth, and age-related natural deepening of the bite are other factors that might have an impact on this condition.

Also known as “acquired deep bites”, deep bites are mainly brought on by environmental factors. It is a well-known fact that there is a dynamic equilibrium of forces between the structures around the teeth, specifically the tongue, the buccinator, the mentalis, and the orbicularis oris muscles, and the occlusal forces that help in the balanced development and maintenance of the occlusion. A malocclusion can be caused by any environmental factor that upsets this dynamic balance; the instances include:

1. A tongue protrusion to the side or an improper tongue position that causes the back teeth to be infraoccluding;
2. Abrasive tooth wear or erosion of the occlusal surface;
3. The posterior teeth’s anterior tips pointing toward the extraction sites;
4. Continually sucking one’s thumb.

To create a thorough diagnosis and treatment plan for each patient and achieve the best possible skeletal, dental, and esthetic outcomes, it is necessary to investigate the etiology of deep bites carefully.

### 1.2. Deep Bite Prevalence

Increased overbite, or deep bite, is defined as a vertical overlap of the incisors that is perpendicular to the occlusal plane and is quantified in millimeters (mm), proportionately (incisor overlap %), or subjectively (lower incisor contact with upper arch or palate). According to Nielsen (1991), skeletal origins of deep bite (low mandibular plane angle, decreased lower face height) and dentoalveolar origins (overeruption of the teeth) are the two most typical categories [15]. According to threshold values used, ethnic group, and gender, deep bite prevalence ranges from 8.4 to 51.5% and 5.9 to 15.9% of cases of palatal impingement and non-traumatic tooth contact have been documented [16,17,18]. Comparing Class II malocclusion to Class I malocclusion, Lux et al. (2009) found a strong correlation between the two and higher overbite [18]. A deep bite may be connected to Class II Division 2, which has a prevalence of 5.3% and is a less common malocclusion [19]. According to the literature, Upadhyay et al. (2008), angle Class I and II Division 2 malocclusions both exhibit an elevated overbite in conjunction with retrusive incisors [20].

## 2. Methods

### 2.1. Literature Search for Research on the Genetics of Deep Bite Development

The 2009 checklist, the GRADE criteria, and the PRISMA recommendations for systematic meta-analyses and reviews were all followed in this investigation. We searched for articles that discussed the genetic or epigenetic components of deep bite between the early 1990s and May 2023. We identified studies that met the following inclusion criteria: (1) original study or meta-analysis; (2) English-language writing; (3) deep bite in humans; (4) genetics of deep bite in humans; (5) prevalence of deep bite in humans; and (6) diagnosis and treatment of deep bite in humans. The following studies were disqualified from consideration: histopathologic, in vitro, or computational studies; transcriptomic or expression studies without epigenetic/genotyping analysis; reports focusing on other conditions and malocclusions that were merely discussed; and reports for which we lacked access to the full text or that were written in a different language. Using the search engines PubMed (National Library of Medicine) and Google Scholar (https://scholar.google.com, accessed on 1 April 2023), a study was conducted in the months of April and May 2023 using the terms “human deep bite”, “genetics of human deep bite”, “prevalence of human deep bite”, “diagnosis of human deep bite”, and “treatment of human deep bite”. Three authors independently evaluated the titles and abstracts, evaluated the database search results, and considered carefully examining the work. Any disagreements were resolved through consensus during the title/abstract or complete article review rounds. Each qualifying study in this systematic review was formally assessed. Separately evaluated by the authors were the included studies’ quality evaluation and bias risk.

### 2.2. Clinical Records and Ethical Statement

The demographic and clinical information for the analysis is obtained from the patient’s orthodontic records. The gender, date of birth, age at treatment initiation, suggested treatment regimen, including extraction and non-extraction of premolars, and length of active orthodontic treatment. To estimate total treatment duration, the starting date is defined as the date of first molar band placement or first direct bonding, and the completion date is defined as the date of orthodontic retainer delivery. All clinical photos were obtained after signing a consent form by the patients to access their data.

### 2.3. Growth Considerations

There is general agreement that treating patients who are still developing makes correcting their deep bite easier and more stable than treating individuals who have stopped growing significantly [21]. It is generally beneficial to treat such patients at a phase of vigorous mandibular growth since growth tends to increase the vertical relationship between the maxillae and the mandible. Because condylar growth enables dentoalveolar growth during the growing period, tooth eruption can be induced in the posterior segments but repressed in the anterior. Even more so in a patient with a hypodivergent skeletal arrangement, the posterior occlusion prevents such a movement in adults. If such a tooth movement is carried out, its stability is really in doubt since it alters the physiology of the muscles, which raises the risk of recurrence. Fixed or removable appliances are required to produce the best treatment outcomes in these malocclusions and others where growth stimulation is no longer viable. Surgical intervention can be necessary for some patients with severe skeletal deformity.

### 2.4. Assessment of the Vertical Dimension

Varlık et al. (2013) recommended incisor intrusion as the best treatment for deep bite correction [22]; however, Mapare et al. [23] and others decided to treat the bulk of their patients with premolar and molar eruption. Instead of relying solely on anecdotal evidence, it is crucial to carefully analyze how extrusive or intrusive mechanics may change a patient’s vertical facial height, which may then have an impact on how the maxilla and mandible are related anteriorly and posteriorly.

The interocclusal space, often known as the freeway [24], is the space between the occlusal or incisal surfaces of the mandibular and maxillary teeth when the jaw is in the physiological rest position. Between 2 and 4 mm is typical. More incredible options for correction occur when there is a larger-than-normal free space because vertical alveolar development can be guided. Increasing the lower facial height or face convexity, for instance, can cure the deep bite and enhance facial esthetics in Class II, Division 2 patients with a hypodivergent facial pattern, redundant lips, and a flat mandibular plane angle. The point A–point B discrepancy and an abnormally broad lower face would be accentuated in most other Class II malocclusions, though, and increasing the vertical dimension is not necessarily desirable in these cases.

### 2.5. Soft Tissue Evaluation

For deep bite correction in the modern era, “soft tissue relationships” are a critical diagnostic tool. When deciding whether to keep, intrude, or extrude the maxillary incisors relative to the upper lip, the clinician must always take the location of the teeth concerning the position of the lips into consideration. Dynamic smile analysis is becoming more popular than static grin photography for evaluating malocclusions and creating effective treatment regimens for their repair as the emphasis on smile esthetics and smile design grows [25].

**Figure 6:**
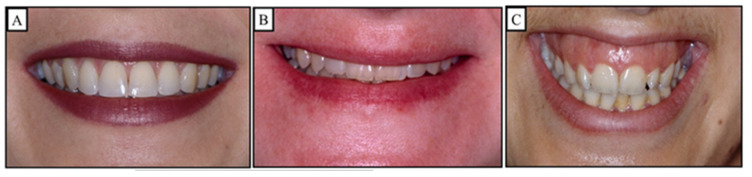
The different types of smiles. (**A**) shows an “Average smile”; the typical incisor exposure is 75–100% of the front tooth length. (**B**) shows a low smile; the typical incisor exposure is less than 75% of the front tooth length. (**C**) shows a “high smile”; the typical incisor exposure is more than 100% of the front tooth length with the appearance of the gingiva more than 2 mm.

**Figure 7:**
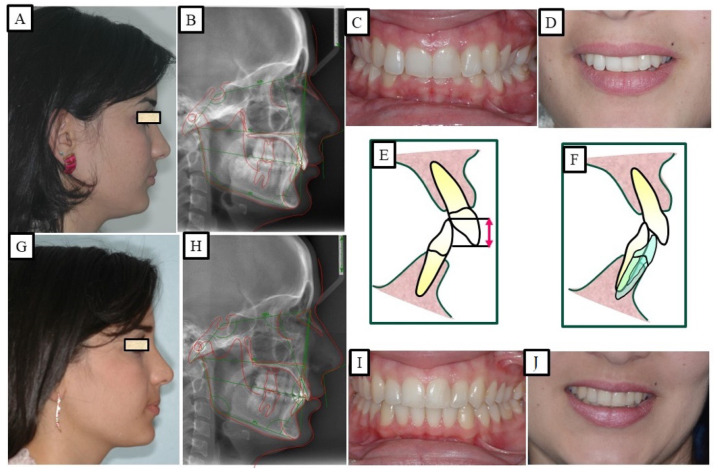
A patient with a deep bite. The treatment was achieved via the intrusion of the mandibular anterior teeth and simultaneous extrusion of the posterior teeth. (**A**–**E**) show before orthodontic treatment and show an average smile; therefore, the intrusion was performed in the mandibular anterior teeth. (**F**–**J**) show after orthodontic treatment.

**Figure 8:**
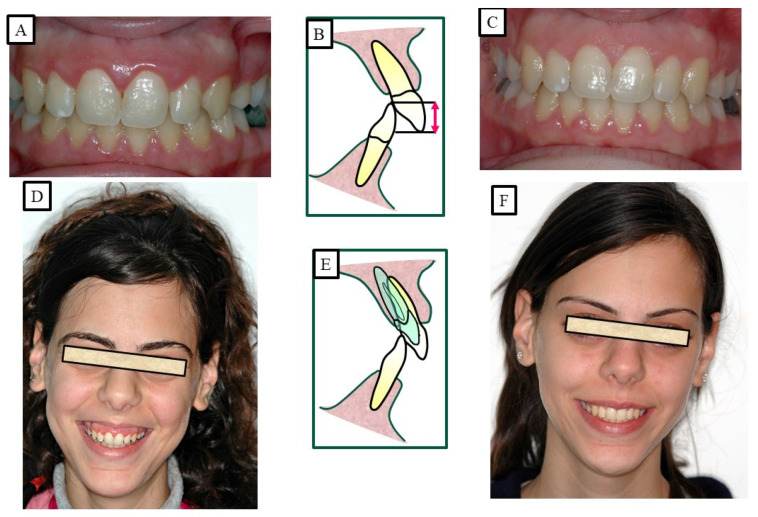
A patient with a deep bite and gummy smile, and the treatment was achieved by the intrusion of the maxillary anterior teeth. (**A**–**C**) show before orthodontic treatment, where the patient showed a high smile; therefore, the intrusion was performed in the maxillary anterior teeth. As a result of the intrusion and minimal gingivectomy, the gammy smile was significantly reduced or eliminated. (**D**–**F**) show after orthodontic treatment.

During the initial assessment, the possibility of incisal exposure should be taken into account in three distinct clinical settings: speaking, a smile, and a relaxed lip position. An acceptable amount of incisor exposure in a relaxed lip position is between 2 and 4 mm, including the incisal margins. In a smile, the typical incisor exposure is about two thirds that of the upper incisor, according to Drummond and Capelli (2016) (Figure 6). Additionally, they stated that while females may have 1 to 2 mm of gingival exposure, most men’s grins do not reveal any gingiva on the upper lip. The treatment strategy should concentrate on either posterior extrusion (if the vertical parameters permit it) or lower incisor intrusion if this criterion is met and a deep bite is still present (Figure 7). A selective intrusion of the upper incisors may be necessary if the occlusal plane is “significantly” below the ideal since this would display excessive gingiva (Figure 8). Since several separate facial muscles are used during the speech, incisor exposure may provide additional information. The “interlabial gap” is a crucial additional consideration. It would not be advisable to perform posterior extrusive mechanics on patients with a significant interlabial gap since this could make the patient’s appearance worse by widening the interlabial gap. A widening of the interlabial gap can lead to several additional issues, including the inability to close the lips naturally and related functional issues. Comparably, posterior extrusive mechanics may be preferable in people who have redundant upper and lower lips or no interlabial gap but have an extreme overbite.

The incisors can also be flared (proclinated), which essentially hides deep bites. Patients who were born with retroclined incisors (Class II, Division 2 cases), for example, benefit from it the most. To reduce the danger of root resorption, gingival recession, and bone dehiscence, quick labial tipping of mandibular incisors must be avoided, particularly on a small symphysis with doubtful labio-lingual breadth of the alveolar bone [26]. Unwanted face esthetics are another possible contraindication.

### 2.6. Clinical Treatment of Deep Bite

**Figure 9:**
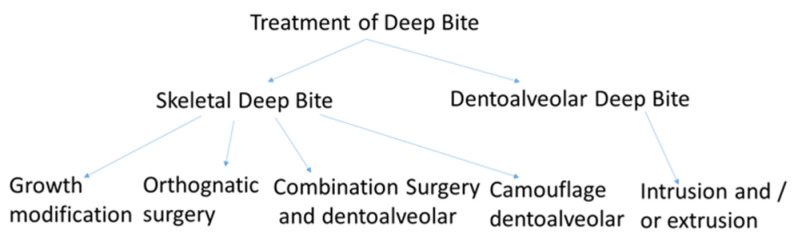
Diagram to illustrate the different types of treatment of deep bite; treatment variants of deep bite depending on age, stage of growth, cause of deep bite, function, and aesthetics.

Several studies have been conducted to investigate the genetic factors that contribute to this malocclusion [27]. The treatment of deep bite depends on various factors, including the severity and type of malocclusion, the patient’s age and overall health, and other individualized considerations. The accompanying treatment approach and several etiologic factors will influence how deep bite correction is administered. However, as was already noted, there are three different techniques to cure deep bite malocclusions: intrusion of the upper and lower incisors, extrusion of the upper and lower posterior teeth, or a combination of the two. Similarly, orthodontic treatment is also the primary treatment for deep bite. The goal of treatment is to realign the teeth and jaws to achieve a more even bite. This may involve using braces, clear aligners, or other orthodontic appliances to move the teeth into the correct position. In more severe cases, orthognathic surgery may be necessary to correct the underlying skeletal issue causing the deep bite (Figure 9).

**Figure 10:**
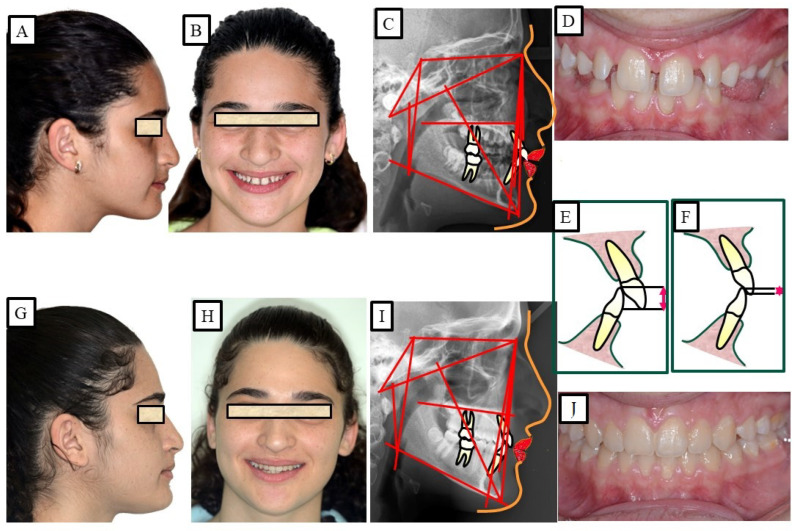
A young patient with dentoalveolar deep bite and average smile. The deep bite is corrected by the intrusion of the front teeth (especially the front of the lower jaw) and the extrusion of the posterior teeth. (**A**–**E**) show the case before orthodontic treatment, while (**F**–**J**) show after orthodontic treatment.

**Figure 11:**
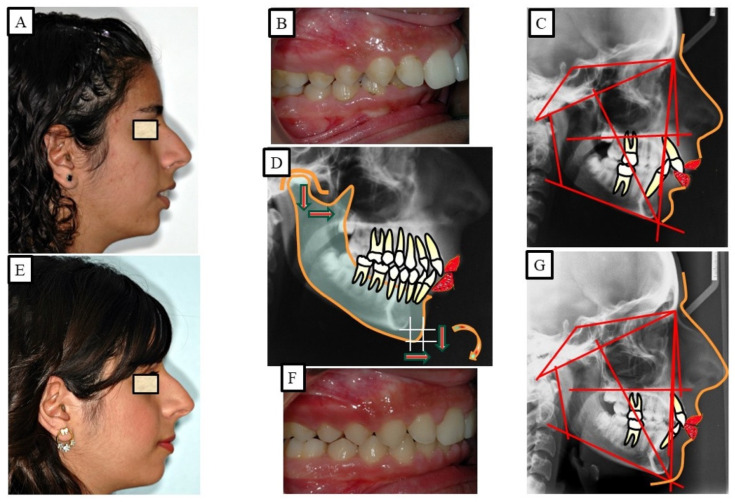
A young patient with Class II dysgnathy and deep bite (skeletal) in growing age. The correction of the deep skeletal bite is performed by influencing growth vertical; the condyle is the growth center. (**A**–**E**) show before orthodontic treatment. (**D**) shows a simulation of mandibular displacement through growth modification, a change in mandibular position due to growth in all dimensions. (**E**–**G**) show after orthodontic treatment.

**Figure 12:**
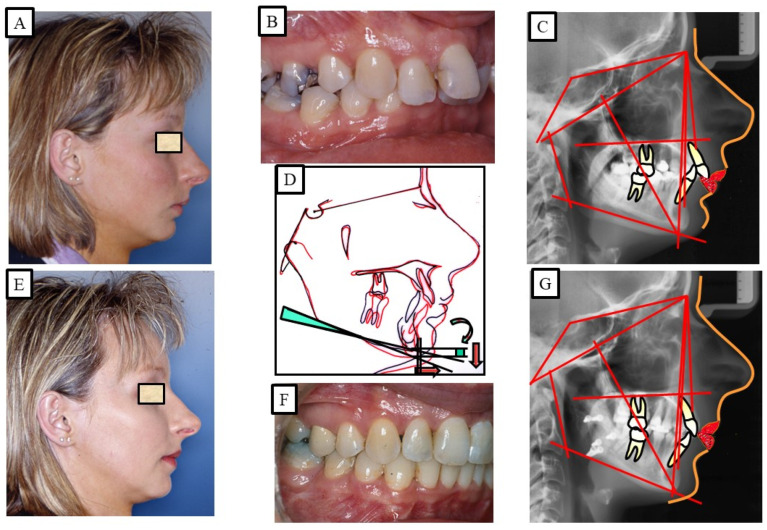
An adult patient with Class II dysgnathy and deep bite (skeletal). The correction of skeletal Class II and the skeletal deep bite is performed via combined orthodontic surgical treatment. Posterior rotation of the mandible during surgical mandibular advancement caused bite elevation and lengthening of the lower facial height. (**A**–**C**) show orthodontic surgical treatment. (**D**) shows the over-positioning of the pre-treatment (black) and post-treatment (red) radiographs due to the changes in sagittal and vertical dimensions. The surgical rotation of the mandible caused the opening of the mandibular angle, which led to the lengthening of the lower facial height. (**E**–**G**) show after orthodontic treatment.

**Figure 13:**
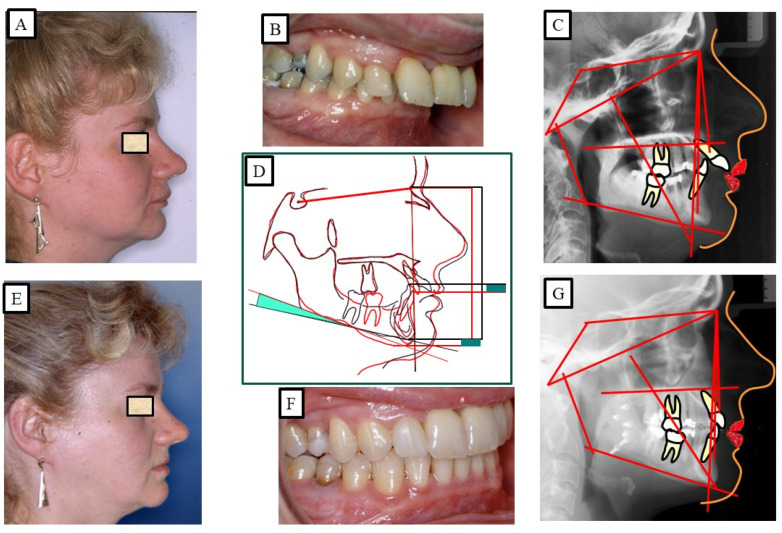
An adult patient with Class II dysgnathy and deep bite (skeletal and dentoalveolar). The correction of skeletal Class II and skeletal deep bite is performed via intrusion of the frontal teeth and combined orthodontic surgical treatment. Posterior rotation of the mandible during surgical mandibular advancement caused bite elevation and lengthening of the lower facial height. (**A**–**C**) show before orthodontic surgical treatment. (**D**) shows the over-positioning of the pre-treatment (black) and post-treatment (red) radiographs due to the changes in the sagittal and vertical dimensions. The surgical rotation of the mandible caused the opening of the mandibular angle, which led to the lengthening of the lower facial height. (**E**–**G**) show after orthodontic treatment.

**Figure 14:**
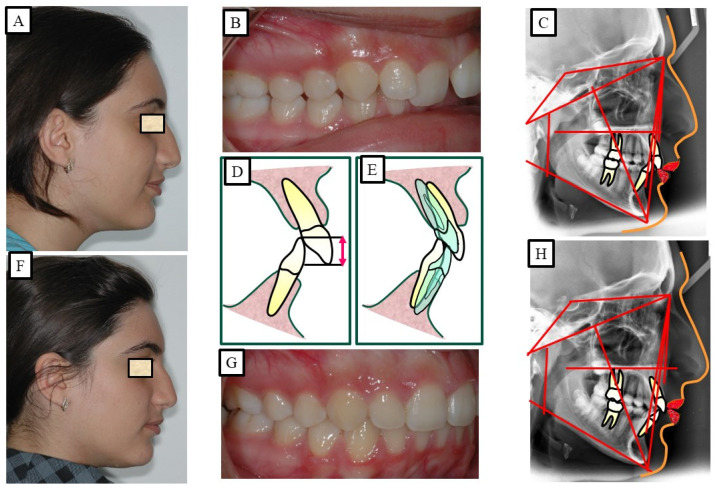
An adult patient with Class II dysgnathy and deep bite (skeletal and dentoalveolar). The correction of skeletal Class II and the skeletal deep bite is performed via camouflage therapy, intrusion and protrusion of the frontal teeth, and extraction of the posterior teeth. (**A**–**D**) show before orthodontic surgical treatment, while (**E**–**H**) show after orthodontic treatment.

According to Proffit and Fields [17], deep bites are the most typical malocclusion in children and adults. “Overbite more than 5 mm is found in nearly 20% of the children and 13% of the adults”, claims [17]. If the patient does not want repair for aesthetic reasons, subjects with minor deep bites usually do not need to be corrected. Although it is a clinical issue, a significant overbite should be treated with orthodontic or orthosurgical intervention. According to Amarnath et al. [28], a severe overbite can damage the incisive papilla, wear down the teeth, disrupt mastication, affect the temporomandibular joint, and wear down the gums. A deep bite can be corrected using a variety of orthodontic techniques. The etiology of malocclusion and an analysis of the critical variables must be considered while choosing a treatment plan for each patient, as presented in Figure 10, Figure 11, Figure 12, Figure 13 and Figure 14. One of the most significant issues facing orthodontists is the maintenance of a deep bite that has been repaired. Relapses following treatment are frequent when appropriate etiologic factor identification is not performed.

Moreover, technological advancements, such as 3D printing and computer-assisted design and manufacturing (CAD/CAM), are promising in treating malocclusion. These technologies for facilitate the creation of customized orthodontic appliances and surgical guides, thereby improving treatment precision and efficiency. Overall, a personalized treatment plan developed by a qualified orthodontic specialist is essential for achieving the best possible outcomes for the patient. Close consideration of the patient’s age, overall health, and the severity and type of malocclusion is vital for successful treatment. Therefore, open communication between the patient and the orthodontist is crucial to create a plan that suits the patient’s unique needs and expectations.

### 2.7. Utilizing Mouse Models and Collaborative Cross Populations to Explore Phenotypes of Deep Bite

Dental conditions such as deep bite can significantly impact an individual’s oral health and quality of life. Exploration the underlying genetic and environmental factors contributing to these conditions are crucial for developing effective treatment strategies. Mouse has shown similar vulnerability to numerous infections and environmental factors to humans; therefore, many restrictions in studies of human populations can be overcome. Mouse models and collaborative cross populations have emerged as valuable tools for studying complex traits and uncovering the intricate mechanisms involved in dental disorders. This article explores how these innovative approaches can shed light on the phenotypes of deep bite, offering new insights into their etiology and potential therapeutic interventions [29].

Unveiling the Genetic Basis Mouse models provide a powerful platform for investigating the genetic factors contributing to deep bite. By manipulating specific genes or introducing mutations, researchers can create mice with dental phenotypes resembling these conditions. Studying these models allows researchers to identify candidate genes involved in the development and maintenance of dental occlusion. Furthermore, by utilizing knockout or knock-in mouse models, scientists can investigate the effects of specific genes and their interactions on dental morphology and occlusal relationships [30].

Standard laboratory mouse lines, however, contain little genetic variety and are therefore only marginally relevant for researching diverse genetic manifestations within complex disorders. To address this, the collaborative cross (CC), genetically varied recombinant inbred mouse lines were created. The CC mouse lines were developed to be an emerging technique for precise genomic mapping and characterization of the genetic components behind complex phenotypes, focusing on critical importance to human health. The requirement to simulate genetic diversity led to the formation of the mouse CC genetic reference population (GRP). This one-of-a-kind GRP source is a large panel of recombinant inbred (RI) strains created particularly for complex trait research from a genetically heterogeneous group of eight founder breeds [7,8,10] suggesting a strength over any previously reported approach [9]. This unique resource is a large panel of recombinant inbred (RI) strains derived from a genetically diverse set of eight founder strains and designed specifically for complex trait analysis [9,31], and suggests a power than any reported approaches earlier [32,33,34,35]. The founder strains are genetically varied, comprising three wild generated strain founders (CAST/Ei, PWK/PhJ, and WSB/EiJ) and five common laboratory strains (A/J, C57BL/6J, 129S1/SvImJ, NOD/LtJ, and NZO/HiLtJ). The substantial genetic variation of the final group of CC mice is a result of this divergence. An entirely different genetic mosaic can be created in a new CC line by altering the sequence of the founder strains during the outbreed mating stage. As a result, each CC line’s genetic component is distinct and has genotypes that are stable and well known. Compared to previous mouse sets, this genetic reference population (GRP) contains a comparatively high degree of recombination events (4.4 million SNPS segregate between the founders), two times the number of genetic differences present in the normal human population (about 36 million SNPs). The latest QTL assessment stimulation research utilizing the CC population revealed that the mapped interval’s resolution may be less than one Mb [32,33,34,35].

Collaborative cross (CC) populations offer a unique opportunity to dissect the complex nature of deep bite. The CC is a panel of genetically diverse recombinant inbred mouse strains derived from multiple founder strains. This diversity enables researchers to study the effects of genetic variation on phenotypic variability. By phenotyping the dental occlusion of CC mice, researchers can identify genetic loci associated with deep bite traits. Subsequent mapping studies can pinpoint specific genomic regions and candidate genes contributing to the observed phenotypes, facilitating a deeper understanding of the underlying biology [36]. In addition to genetic factors, environmental influences play a critical role in developing dental occlusion abnormalities. The CC mouse genetic reference population (GRP) allows researchers to investigate gene–environment interactions by subjecting CC mice to various environmental conditions or exposures. By manipulating factors such as diet, mechanical loading, or hormonal influences, researchers can evaluate how these external variables interact with genetic predispositions to affect dental occlusion. This multifaceted approach provides insights into the complex interplay between genetic and environmental factors in the development of deep bite and open bite.

It should be possible to run GWAS on CC breeds, identify crucial quantitative trait loci (QTL), discover candidate genes, and define modifiers for the key genes linked to the Deep bite features while under minimal levels of external sources of variance. It is strongly believed that the tremendous genetic diversity of the CC mice strains offers a good foundation for finding novel genetic loci connected to these described traits and going forward with confirmation utilizing conditional knockout techniques and mouse knockout genes.

**Figure 15:**
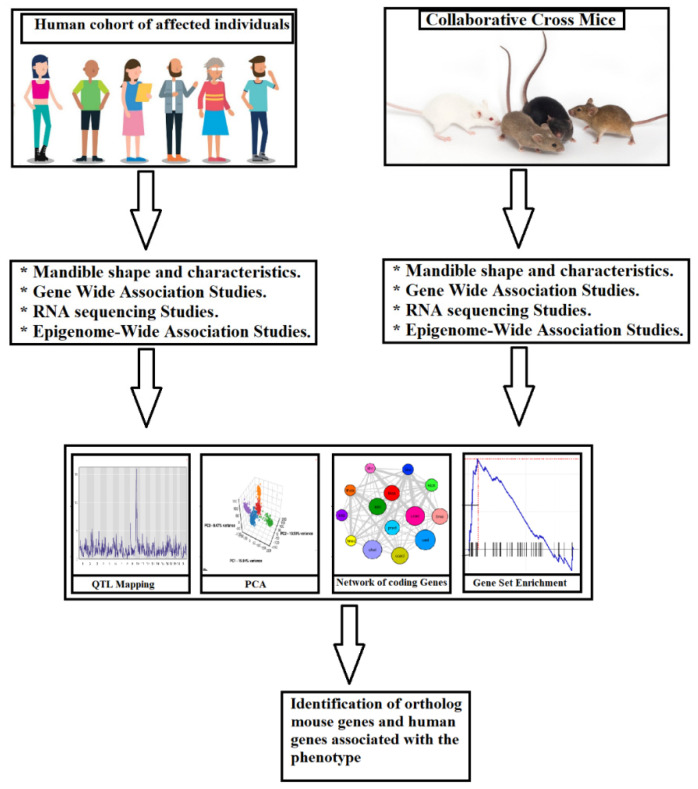
Workflow for creating system genetic databases from the extremely heterogeneous CC population, whose propensity to develop deep bite malocclusion may vary greatly. To check for deep bite malocclusion, mice are examined. Then, different associations between malocclusion and deep bite characteristics are analyzed using a combination of cellular, molecular, and clinical trait data. The regulatory genomic areas are implicated in phenotypic variation in both in vitro and in vivo monitored traits and can be identified using QTL mapping by merging SNP genotype data from each CC lineage. Finding susceptibility genes linked to the emergence of deep bite in humans may be possible by combining data with later candidate gene association studies in humans.

The knowledge gained from mouse models and CC populations can be translated into human dentistry, improving diagnostic and therapeutic strategies. Understanding the genetic and environmental factors underlying deep bite in mouse models enables researchers to identify potential biomarkers and genetic risk factors in humans. These findings may inform the development of targeted interventions and personalized treatment approaches. Furthermore, mouse models allow for preclinical testing of novel therapeutic interventions, such as gene therapies or pharmacological treatments, before translating them into clinical trials [29,37]. The workflow diagram for the generation of system genetic datasets of cellular, molecular, and clinical trait data combined to analyze various correlations between malocclusion and deep bite phenotypes in human and mouse models are represented in Figure 15.

## 3. Discussion

Excessive vertical overlapping of the mandibular incisors by the maxillary incisors in a centric occlusion is also known as a deep bite or deep overbite. The normal overbite is between one and three millimeters, and the incisal margins of the lower teeth should touch the upper teeth’s cingulum or just above it. According to Sreedhar and Baratam (2009), the average overbite is roughly 30%, or one third, of the mandibular incisors’ clinical crown height because of variations in their length [38]. Numerous researchers have examined the skeletal and dental patterns associated with deep bite malocclusion. Deep bite malocclusion was found to be associated with a decreased gonial angle, a deep curve of the spee, a smaller posterior maxilla, a downward rotation of the palatal plane, and a more forward position of the ramus, according to research by Fattahi et al. (2014), who examined the morphologic factors in deep bite and patients [14]. Alveolar and skeletal dimensions related to overbites and reduced facial height were evaluated by Beckmann et al. [39]. They hypothesized that a deeper bite was associated with decreased lower facial height, bigger anterior alveolar and basal regions, and retroinclination of the maxillary incisors [39]. In their 2004 study, Bydass et al. examined how overbite and overjet were affected by the depth of the spee curve. Because of the lower anterior teeth that protruded, there was an increase in overbite in the deep curve of the spee [40]. According to Ceylan and Eroz (2001), an overbite can change how the mandible and maxilla look and is linked to a smaller gonial angle [41]. In participants with a deep bite and a normal overbite, Al-Zubaidi and Obaidi (2006) measured the lower facial height (LFH) [42]. They discovered no variations in the LFH, maxillary and mandibular anterior alveolar, and basal height between the two groups. El-Dawlatly et al. (2012) assessed skeletal and dental parameters in patients with deep bite malocclusion. They demonstrated that deep bite has a multi-factorial etiology, with the exaggerated curve of spee and a decreased gonial angle being the main contributing factors [43]. Naumann et al. (2000) investigated the vertical elements of overbite alteration in a longitudinal study. Their study revealed that skeletal elements had a more significant impact on overbite modification than dental elements and that the mandible had a more significant impact on overbite modification than the maxilla [44].

According to earlier research, the maxillary dentoalveolar region is where a deep bite and a typical bite vary. Dentoalveolar morphology of the upper and lower jaws, according to Betzenberger et al. [45], was the cause of overbite alterations. This study aims to identify the most common dental and skeletal contributing variables to deep bite malocclusion and the effects of skeletal and dentoalveolar characteristics on deep bite malocclusion. Undoubtedly, practitioners are better equipped to provide the most effective care when fully aware of the dental and skeletal causes of deep bite malocclusion [45].

To enable any future reconstructive dental surgery, reduce increased tooth wear, and lessen tissue stress from tooth contact, treatment of deep bite malocclusion is advised [46]. When malocclusion returns years after the conclusion of treatment, patients may request a second opinion or start to doubt the value of their previous therapy. Thus, stability over the long run appears to be more crucial than the actual outcome. Even in cases that have received the best care possible, relapse—a dentoalveolar and skeletal change that occurs after orthodontic treatment and returns the mouth to its original malocclusion—is frequently seen [47]. These alterations are attributed to natural restoration of force homeostasis [17], periodontal remodeling [48], growth, or normal/abnormal development (Iseri and Solow (1996)). Some investigators (Al Yami et al. (1999)) found a constant relapse of all malocclusion characteristics and the loss of around one third of the orthodontic treatment outcome over a ten-year period of follow-up [49]. Thus, one of orthodontics’ greatest challenges is maintaining the stability of the orthodontic outcome.

Dental deep bite cases are said to have relapsed if their overbite increases after therapy is complete. Deep bite malocclusions are said to be prone to relapse in a number of writers [50]. A sample with a deep bite and retroclined incisors was evaluated by Lapatki et al. (2004), who discovered 20% vertical relapse on average two years after treatment. In research examining samples with different malocclusions, a number of conclusions involving deep bite relapse are documented [51]. A total of 21 out of 31 cases in which the spee curve in a Class II Division 1 sample was examined showed a steady relapse over the years or decades [52]. In a similar vein, a Class II Division 2 sample from Canut and Arias (1999) discovered a positive connection between years out of retention and overbite relapse [53]. Despite using removable retention for a year, 80% of patients with short facial types experienced a rise in overbite 2 years after treatment was finished [54].

Growth [55], function [56], and incisor overeruption [57] are some of the factors that might contribute to the formation of a deep bite and may also do so in the case of a relapse. Pre-treatment severity of malocclusion and relapse were not significantly correlated, and mandibular intercanine width, overbite, overjet, mandibular incisor irregularity, or arch length were not able to predict relapse, according to Preston et al. [58]. No matter the method of treatment, the authors discovered a much higher prevalence of relapse in patients whose dentitions had not fully leveled at the end of the procedure. Different relapse rates regardless of the method of treatment were not demonstrated by a number of authors [58]. An overbite relapse was linked in one research to mandibular incisors that protruded after orthodontic treatment. It is currently not possible to anticipate an individual’s risk of relapse following deep bite therapy because there is neither a systematic review of deep bite retention, stability, or relapse, nor is there any way to determine if a person would experience either.

A significant overbite is one of the issues that orthodontists address the most commonly. According to Sonnesen and Svensson [59], deep bite has been linked to aberrant mandibular function, TMJ issues, and may harm mandibular development. Horiuchi et al. [60] have identified a similar association. Because of this, correcting a deep bite is frequently a crucial part of orthodontic care. It is commonly acknowledged that correcting a deep bite on a patient who is still growing is easier and more stable than trying to do it on someone who has stopped growing significantly [61]. Invasion of the occlusal highway and the correction’s opposition to the robust, mature jaw musculature, which is less adaptable to elongation, have both been identified as causes for the greater relapse potential in adults [62]. Additionally, any tooth movement affects the functional equilibrium that is established during growth and maturity [63]. Through the process of growth and development, the skeletodental and soft-tissue components structurally adapt to one another to create a functionally balanced condition [64]. A shift in the mandibular muscular balance takes place because widening the bite is typically performed via protrusion of posterior teeth [13]. If the correction is to stay stable, either the musculature must adjust in some way to its new functional resting length or the bone arrangement must alter. There are various places in the mandible that have been proven to be able to respond to environmental challenges; it is possible that compensatory development will occur in these sites.

After receiving orthodontic treatment for a moderate deep bite, there was little evidence of vertical relapse 12 years later. If the incisor overlap increased by more than 50% during the follow-up, it was considered a relapse. At long-term follow-up, 90% of the patients displayed normal vertical relations, while 10% of them displayed relapse with a modest median increase of 6.7%. The prevalence and severity of deep bite relapse were relatively low and clinically negligible in instances with mild dentoalveolar deep bite that had undergone effective treatment, retention by fixed retainers, and a temporary removable upper plate.

When facial types were evaluated, a recent study indicated that individuals with high angles had a decreased propensity to relapse than patients with normal or low angles [65]. The relatively lengthy treatment period suggests that the majority of the periodontal remodeling [66] had already occurred at the time of debonding, and it is not anticipated that it would have significantly contributed to the relapse. Treatment options include fixed appliances with or without extraction, removable appliances with or without extractions, and in more severe cases, maxillofacial surgery to treat the deep bite [67]. There have been numerous therapeutic approaches and combinations employed, including maxillofacial surgery, as presented in Figure 12 and Figure 13. There are three possible ways to level the arch/curve of spee and treat deep bite malocclusion with orthodontics: (1) lower and/or upper incisor intrusion [68]; (2) labial inclination of the incisors (pseudo-intrusion); and (3) extrusion of posterior teeth possibly associated with a clockwise rotation of the mandible, which would increase lower face height [47]. According to Bernstein et al. [52], this hypothetical clockwise rotation does not always appear to take place. The available literature cannot be used to draw any conclusions about the efficacy of treating Class II Division 2 malocclusion in children [69]. Different treatment approaches and combinations were used to stabilize deep bites.

The development of skeletal structures is somewhat influenced by the environment and partially by genetics, as this article has shown. It is therefore impossible to discount the significance of the hereditary foundation of malocclusions. The practice of genetically supported orthodontics has advanced significantly. However, because most malocclusions and dental malformations are polygenic, it is very difficult to identify the hereditary basis of these conditions. The mapping of inherited conditions pertaining to dentofacial development has been possible because to the information provided by the human genome project. To accurately identify all the unique genes responsible for each type of skeletal diversity, additional genetic research is necessary. A genetic correction of genetically regulated abnormalities and malocclusions may be possible in the near future due to the field’s rapid advancement.

## Data Availability

Not applicable.
